# Rebirth of *JCHIMP*

**DOI:** 10.3402/jchimp.v3i3-4.23202

**Published:** 2013-12-17

**Authors:** Robert P. Ferguson

This is the third and final issue of volume 3 of *JCHIMP*. It is 3 months later than planned. This is not as serious as with paper journals (except, perhaps, to the waiting authors).

Some Internet open-access journals publish individual articles as they are accepted. When we organized *JCHIMP* in 2010, we made the decision to publish quarterly because of editorial preferences and input from other teaching faculties. Even though this delayed the publication of some manuscripts by a few months, it was still much faster than most paper manuscript processes.

This publishing style was one of the many decisions that were required as we organized the journal. Our intent was to provide an efficient outlet for scholarly work produced primarily in community hospital internal medicine residency programs. We had no illusions about financial profitability. Keeping expenses under control and developing support from hospitals and programs was our goal.

David Solomon was our guide (1). He was the former editor of *Medical Education Online* and had extensive experience with this media. He referred us to Co-Action Publishing in Stockholm, Sweden. They have been outstanding in every respect, fulfilling their obligations, and at the same time, keeping costs down.

Long-term *JCHIMP* was intended to fill a void caused by the demise of many regional paper journals (2). It fit in nicely in the context of the proliferation of Internet journals worldwide. We were particularly interested in serving as a scholarly platform for the Community Hospital Education and Research Network (www.cherninfo.net), CHERN, an organization formed 2 years earlier from the community hospital subgroup of the Association of Program Directors in Internal Medicine (APDIM). We planned to seek out authors and peer reviewers from this constituency. We would review and publish the scholarly activities of community hospital programs, including opinion-oriented perspectives, original research (including observational studies), case reports, and columns of interest such as radiologic and human imaging, electrocardiography, medical education innovations, and the history of medicine. This technology would allow us to link to video images (3).

After the first year, we were very pleased with the readership reaching 2,500 ‘unique individuals’ from 88 countries. The readership and notoriety accelerated since then with over 15,000 different readers from 143 countries by October 2013 – mid-third volume. By issue # 10 (volume 3, # 2), 92 manuscripts had been published. One hundred and forty-one peer reviewers have participated; 88 this year (Table 1). Many of these peer reviewers had little experience in this process prior to *JCHIMP*. We have included residents and medical students and have helped to train them in peer review (4). As the editor, I tailor manuscript assignments based on my knowledge of the reviewer's experience. Some medical students may make their primary review contribution through their previous experience as English majors. All reviews are multiple and we try to mix reviewer experience in a sensible way.

**Table 1:** *JCHIMP* reviewers in 2013

Carlos Acuna	Melissa Delong	Anil Kumar	Mohsen Saadat
Majd Alghatrif	Stephanie Detterline	Jeff Larochelle	Sonia Samtani
Tarek Alansari	Oner Dickensoy	Srinivasa Madhavan	Fardad Sarabchi
Chuck Albrecht	Emmanuel Elueze	Naba Mainali	Andrew Schuldenfrei
Nadia Ali	Robert Ferguson	Mohammad Malik	Chuck Seelig
Richard Alweis	Matthew Ferris	Henry Meilman	Mansur Shomali
Raja Ayash	Negar Forough Saeid	Marita Mike	Peter Sloane
Hooman Bakhshi	Paul Foster	Hmu Minn	David Smith
Shadi Barakat	Steven Gambert	Dimtra Mitsani	Bishnu Subedi
Jennifer Blackman	James George	Robabeh Mohammadzadeh	Lisete Teixeira
Kenzie Bowen	Mohammadali Habibi	Mahsa Mohebtash	Linda Thomas
Dolores Buscemi	Noah Hoskins	Marc Mugmon	Jeffrey Vieira
Wayne Campbell	Pavan Irukulla	Faranak Najibi	Radhika Vij
Harjit Chahal	Yan Ji	Ali Ozhand	Yue Wang
Issam Cheikh	Ning Jin	Zahra Pakbaz	David Weisman
Malek Cheikh	Aditya Kalakonda	Venkataraman Palabindala	Ashley Wietsma
Chester Choi	Joel Katz	Phil Panzarella	David Widlus
Dobbin Chow	Maryam Keshtkar Jahromi	Raj Parekh	Frederick Williams
John Cmar	Bassem khalil	Khalid Qazi	Richard Williams
Candice Crichlow	Robert Kornberg	Celeste Quianzon	Amanda Wong
Stefan David	Mahesh Krishnamurthy	Ashish Rana	Maryellen Woodward
Janaki Deepak	Sapna Kuehl	Nicholas Risko	Jeff Zapora

## HIATUS

In July, 2013, there was a *JCHIMP* hiatus. Circumstances stalled *JCHIMP*. I had decided to move to Greater Baltimore Medical Center. As founder, editor, and chairman of the editorial board, this dictated a restructuring in the financial support we had received from Medstar Union Memorial Hospital since 2010. The editorial process stopped while awaiting resolution; all except manuscript submissions, which continued unabated.

Ironically, it was during this month that the journal received notice from PubMed via our publisher that we were now ‘indexed’. All 92 papers published since volume 1, # 1 were retroactively indexed. This was a crucial achievement for *JCHIMP* and Co-Action Publishing in that the current Internal Medicine Residency Review Committee Special Requirements do not recognize non-PMID peer-reviewed publications when assessing program scholarly activity during the accreditation review process (5).

The financial deficit requiring subsidization was *JCHIMP*'s primary problem. There has been criticism in the paper journal literature of the predatory nature of many Internet journals, evolving into profit status organizations, frequently charging exorbitant publication fees (6, 7). In my opinion, there may be greedy and unscrupulous journal managers, but it does not have to be that way. *JCHIMP* is at the other end of that spectrum and is most definitely not profitable. We have charged no more than $350 for a publication fee and most of the invited papers are not charged any fee. This was a consensus decision by the editorial board. One of our beliefs is that any worthwhile submission should be published regardless of the author's ability to pay.

We now have a new financial model. Our goal is to be self-sufficient in the near future. There will be a modest increase in the publication fee. With our indexing status, we anticipate more potential publications, so much so that we plan to increase to six issues a year rather than four. Other tinkering is being done to create revenue enhancement including residency recruitment advertising, special subscription status, and general fund raising.

We wish to thank Medstar and Union Memorial Hospital for the support provided to the journal during its formative years. We welcome my new department of medicine at GBMC as our new benefactor.

Also in this issue are 10 other manuscripts. Two research studies are very relevant to community teaching hospitals and their faculties: one demonstrates the five station resident interview model which can potentially improve efficiency without sacrificing reliability in the laborious residency application process (8); the other, direct observation of PGY 1's in the summer of 2013 interviewing new admissions, often overestimating patient health literacy, particularly in those where English language is not their first language (9). There are three traditional case reports: cryptosporidium gastroenteritis in an immunocompetent host (10), discovery of a congenital vascular anomaly in a 57-year-old (11), and management of multiple cardiac thrombi (12). Radiology imaging is reported from Spain showing a connection between massive pleural effusion and renal failure (13). ECG imaging demonstrates classic Mobitz 1 second-degree heart block caused by Lyme disease (14). We also have three short clinical reports published for visual interests: apparent chemotherapy induced tongue hyperpigmentation (15); cutaneous larva migrans from walking on the beach (16); and classic necrobiosis lipoidica in a diabetic (17).

*Robert P. Ferguson, MD* Editor
